# Impact of high-risk optic disc on central retinal vein occlusion in patients with metabolic disorders

**DOI:** 10.3389/fphys.2024.1424144

**Published:** 2024-08-15

**Authors:** Shancheng Si, Jiateng Lin, Rong Guo, Anming Chen, Yicong Ji

**Affiliations:** ^1^ Eye Center, Beijing Tsinghua Changgung Hospital, School of Clinical Medicine, Tsinghua University, Beijing, China; ^2^ Department of Ophthalmology, Rongcheng Eye Hospital, Rongcheng, China; ^3^ Endocrine Department, Fuwai Hospital, National Center for Cardiovascular Diseases, Chinese Academy of Medical Sciences and Peking Union Medical College, Beijing, China

**Keywords:** central retinal vein occlusion related macular edema, metabolic disorder, high-risk optic disc, central retinal vein occlusion, macular edema

## Abstract

**Purpose:**

To evaluate the impact of high-risk optic disc (HROD) on central retinal vein occlusion (CRVO) in patients with metabolic disorder(s).

**Design:**

Retrospective case-control study.

**Methods:**

A case-control study involving CRVO patients with metabolic disorder(s) was performed. **PART I**. All eligible patients with CRVO were included in CRVO group, and a similar number of patients with metabolic disorder(s) without CRVO were matched by sex, age and blood glucose level in the non-CRVO group. Various parameters were compared between groups. The impact of risk factors associated with CRVO was presented as odds ratios (ORs) and 95% confidence interval (95% CI). **PART II**. All eyes with CRVO that underwent intravitreal treatment (IVT) with a follow-up duration of ≥1 year were divided into non-HROD and HROD groups, and the differences between the two groups were compared.

**Results:**

In **PART I**, a total of 45 and 63 eyes were enrolled in the CRVO and non-CRVO groups, respectively, with a significant statistical difference in HROD (51.16% vs 26.98%, *p* = 0.010) between them. In further multivariate regression analysis, HROD was the independent risk factor for CRVO (OR = 5.036, 95% CI 1.847–13.729, *p* = 0.002). In **PART II**, demographic, follow-up information, treatment, and prognosis showed no significant statistical difference between the two groups (all *p* > 0.05).

**Conclusion:**

HROD was likely to be an independent risk factor for CRVO occurrence in patients with metabolic disorder(s), but it did not affect the treatment and prognosis of CRVO eyes with HROD.

## Introduction

Central retinal vein occlusion (CRVO) is closely related to systemic diseases, especially hypertension, diabetes, and total cholesterol (TC) (Song et al., 2019; Liu et al., 2021). However, in addition to the known relationship between glaucoma and CRVO (Yin et al., 2019; Chen et al., 2021), there are more disputes about the ocular risk factors of CRVO, especially the relationship between ocular anatomical characteristics and CRVO. A recent literature (Lei et al., 2021) reported that five female children with CRVO of unknown cause had the characteristics of a crowded high-risk optic disc (HROD), suggesting a correlation between ocular anatomical characteristics and the occurrence of CRVO in some specific individuals. Chan et al. (2013) believed that branch retinal vein occlusion was related to a larger cup area, larger disc area, and larger vertical cup-to-disc ratio (VCDR) in the Singapore Indian eye study. Similarly, Klein et al. (2006) also found that in the Beaver Dam Eye Study, the incidence of retinal vein occlusion (RVO) increased with an increase in VCDR. Nevertheless, Mansour et al. (1990) and Xu et al. (2012) believed that the anatomical structure of the optic disc had nothing to do with the occurrence of RVO.

However, none of the above studies have explored the impact of HROD on CRVO occurrence in specific individuals with metabolic disorder(s). It was reported that diabetes and dyslipidemia were important risk factors for CRVO occurrence (Song et al., 2019; Liu et al., 2021); And HROD was a risk factor for various ocular vascular diseases, especially anterior ischemic optic neuropathy. Therefore, we speculated that HROD may increase the risk of CRVO occurrence in patients with metabolic disorder(s). In this study, we not only paid attention to the above systemic risk factors of CRVO, but also paid more attention to ocular anatomical characteristics. In other words, we aimed to explore the ocular risk factors (especially HROD) for CRVO occurrence in individuals with metabolic disorder(s).

## Methods

### Study Design and Participants

A retrospective case-control study involving CRVO patients with metabolic disorder(s) was performed at Beijing Tsinghua Changgung Hospital. A total of 108 enrolled participants (45 CRVO and 63 non-CRVO patients) underwent a series of ophthalmic and systemic examinations at our hospital between April 2021 and November 2022. This study was approved by the Institutional Review Board of Beijing Tsinghua Changgung Hospital and was conducted in accordance with the tenets of the Declaration of Helsinki (approval No. 23218-6-01). The Board waived the requirement for written consent because of the retrospective nature of the study. All analyzed data were anonymized and de-identified.

Demographic data (age, sex, laterality, etc.), ocular features (HROD, primary glaucoma, etc.), medical history of metabolic disorder(s), such as maximum systolic blood pressure (SBP), treatment procedures, and prognosis (low vision, blindness, etc.) were collected from the outpatient electronic medical record system at enrollment. Baseline blood parameters, such as glycated hemoglobin A1c (HbA1c), estimated glomerular filtration rate (eGFR), and lipids, were collected. Color fundus photography (CFP, TRC- 50DX or Canon CR-2 machines) and optical coherence tomography (OCT, spectral-domain OCT, Heidelberg Engineering, Heidelberg, Germany) without pupil dilation were performed at each visit. The diagnosis of CRVO was based on CFP and central retinal vein occlusion-induced macular edema (CRVO-ME) was based on OCT. Ophthalmologists (SS and JL) reviewed and confirmed the diagnosis. VCDR data was evaluated using OCT prior to CRVO occurrence by the same technician (AC) and was then confirmed by superior specialist (JL), which was masked to the grouping. Primary glaucoma was defined as primary open-angle glaucoma or primary angle-closure glaucoma (PACG). Low vision was defined as a best-corrected visual acuity (BCVA) < 20/60, and blindness was defined as a BCVA <20/400.

### Grouping and definitions

#### Part I

The CRVO group met the following inclusion criteria: All CRVO patients (1) with initial CRVO diagnosis between April 2021 and November 2022 and (2) with at least one previous OCT before CRVO occurrence. The exclusion criteria were as follows: CRVO patients (1) with neither diabetes nor dyslipidemia; (2) without available OCT for measuring the VCDR before CRVO occurrence; or (3) with an axial length of ≥26.5 mm; or (4) with the age of ≤18 years.

The non-CRVO control group matched by sex, age and blood glucose level was derived from patients with metabolic disorder(s) (diabetes or/and dyslipidemia) for fundus screening by CFP combined with OCT in our outpatient department of ophthalmology during the same period, but excluding type 1 diabetes mellitus and a large number of type 2 diabetes patients, including those with diabetic retinopathy of ≥ severe non-proliferative diabetic retinopathy, diabetes duration of >20 years, and HbA1c > 9%. In this study, patients with metabolic disorder(s) were defined as those with type 2 diabetes or dyslipidemia. Dyslipidemia was defined as blood TC ≥ 5.2 mmol/L or blood low-density lipoprotein cholesterol (LDL.C) ≥ 3.4 mmol/L. An endocrine expert (RG) reviewed and confirmed the diagnosis of type 2 diabetes mellitus and dyslipidemia. All patients were of Chinese Han ethnicity, and only one eye of each patient was included in the study (the affected eye in the CRVO group and the right eye in the non-CRVO group).

#### Part II

**FIGURE 1:**
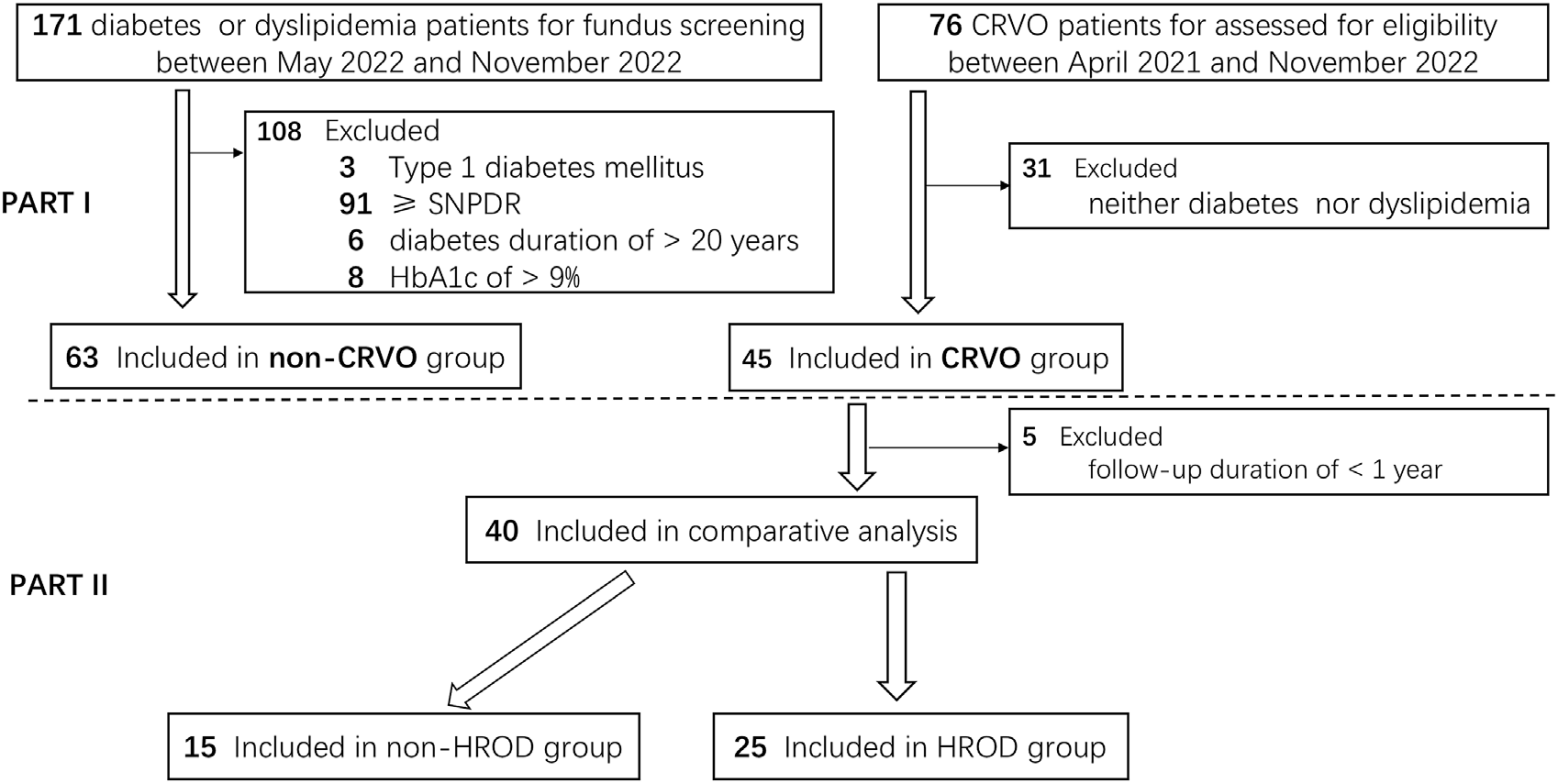
The detailed screening and grouping flowchart of all recruited subjects. CRVO, central retinal vein occlusion. SNPDR, severe non-proliferative diabetic retinopathy.

All patients receiving intravitreal treatments (IVTs) for CRVO-ME with a follow-up duration of ≥1 year and without CRVO-ME recurrence of ≥3 months from the CRVO group were included in **PART II**. The development, treatment, and prognosis differences between eyes with CRVO with and without HROD in **PART II** were then compared. CRVO-ME recurrence was defined as central retinal thickness ≥300 µm and visual acuity <20/30. Detailed screening and grouping of all recruited subjects were shown in Figure 1.

### Definitions of terms and representative photos

**FIGURE 2:**
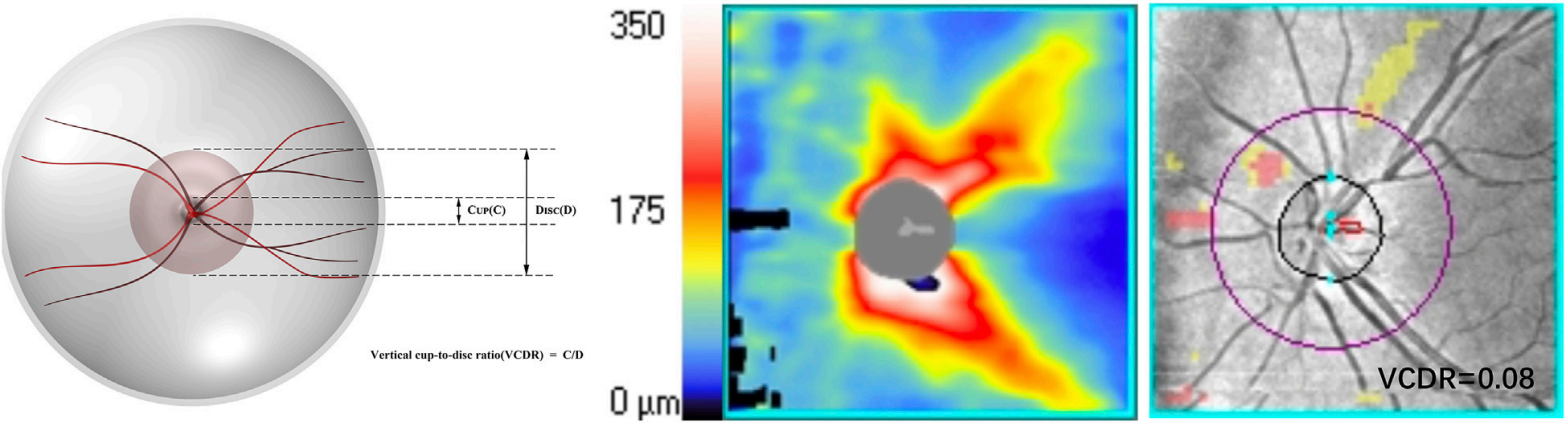
An illustration of the optic nerve configuration and the vertical cup-to-disc ratio (VCDR).

VCDR: a ratio between the vertical diameter of the optic cup and the optic disc (Figure 2).

**FIGURE 3:**
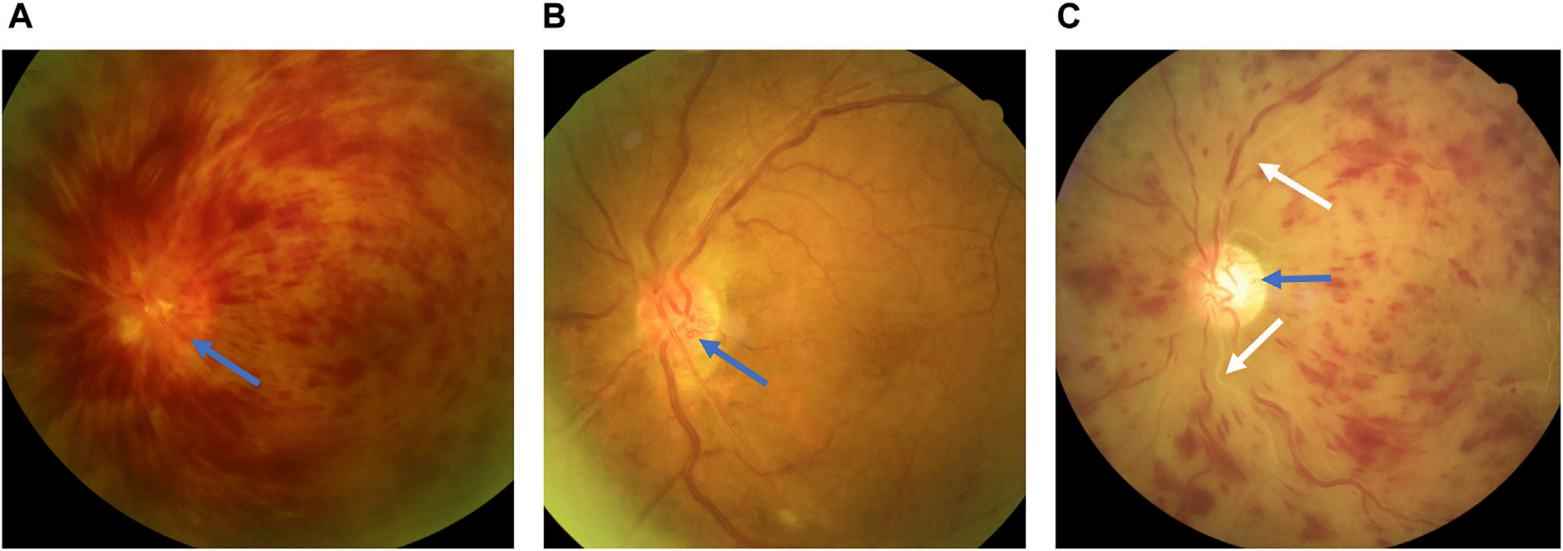
Representative photos used to explain relevant terms. A 64-year-old female was diagnosed with central retinal vein occlusion (CRVO) due to blurred vision in the left eye for more than 2 months, with a 9-year history of type 2 diabetes and grade 2 hypertension. **(A)** The color fundus photography (CFP) at the first diagnosis in April 2019 showed typical flame-shaped hemorrhages and a high-risk optic disc (HROD), with crowded optic disc without optic cup (blue arrow). **(B)** At the reexamination in September 2019, the flame-shaped hemorrhages were completely absorbed, and HROD was more clearly displayed. **(C)** Another 64-year-old male was diagnosed with CRVO in the left eye. In October 2021, the superior and inferior temporal branch retinal arteries (BRAs) all showed complete whiteness (white arrows) of distal branch, as well as proximal trunk, without HROD (blue arrow).

HROD: crowded optic disc without optic cup (Figures 3A, B) or optic nerve configuration with a VCDR of 0.2 or smaller on OCT by at least 2 independent evaluators (Lei et al., 2021).

Complete whiteness of one or two temporal branch retinal arteries (CWTBRAs): the image features on CFP with not only white-line change of the distal branch, but also white-line change of the proximal trunk, involving one or two temporal branch retinal arteries (Figure 3C).

Ocular hypertension (OHT) events within the follow-up period: any event with intraocular pressure of >25 mmHg during the entire follow-up period, including primary open-angle glaucoma, primary angle-closure glaucoma, OHT induced by intravitreal dexamethasone implant, and postoperative and unexplained OHT, but excluding CRVO-induced neovascular glaucoma (NVG).

### Statistical Analysis

All calculations were performed using SPSS Statistics for Windows software (version 25.0, IBM Corp.). Continuous variables were recorded as means and standard deviations (mean ± SD) or medians (interquartile range, IQR) according to whether the data were normally distributed, and categorical variables as counts and percentages. The Shapiro-Wilk test was used to check the normality of the data. BCVA was converted to the logarithm of the minimum angle of resolution (log MAR) units for analysis. For BCVA of counting fingers or worse, the following conversion was used: counting fingers, 2.0 log MAR; hand movements, 2.3 log MAR; light perception, 2.6 log MAR and no light perception, 2.9 log MAR (Niederer et al., 2022).

First, an independent-sample *t*-test was used to compare continuous variables such as demographic and ocular characteristics, metabolic disorder(s), and blood test results if appropriate; the Mann-Whitney U test was used to compare the statistical differences between two groups of hierarchical variables (hypertension grade); and the chi-square test or Fisher’s exact test was used to compare categorical variables. And then, Spearman rank-based correlation analysis was used to calculate the correlation coefficient between baseline features with a *p*-value <0.1 (including ocular features, metabolic variables and blood tests) and CRVO; Pearson’s partial correlation analysis was used to calculate the covariate-adjusted correlation coefficient. Next, the baseline features with a *p*-value <0.10 (HROD and LDL.C) were included in further logistic regression.

Univariate logistic regression analysis was performed to evaluate the potential risk factors (HROD and LDL.C) associated with CRVO. The significant risk factors (HROD and LDL.C) associated with CRVO in the univariate model (*p* < 0.10) were considered potential confounders in the multivariable models, and the results were presented as odds ratios (ORs) and 95% confidence intervals (95% CI). Third, all CRVO eyes in **PART II** were divided into non-HROD and HROD eyes according to whether there was an HROD, and the differences in demography, ocular features, treatment, and prognosis were compared between CRVO eyes with and without HROD. Statistical significance was defined as a 2-sided *p*-value < 0.05.

## Results

### Demographic, ocular and systemic characteristics of all recruited patients with metabolic disorder(s)

In **PART I,** of 76 CRVO patients screened, 31 (40.79%) patients were excluded for neither diabetes nor dyslipidemia. Forty-five (59.21%) of the screened individuals met the study protocol and was included in CRVO group. Of 171 non-CRVO patients with metabolic disorder(s), 108 (63.16%) patients were excluded due to various reasons, including 3 for type 1 diabetes mellitus, 91 for diabetic retinopathy of ≥ severe non-proliferative diabetic retinopathy, 6 for diabetes duration of >20 years, and 8 for HbA1c of >9%. At last, 63 (36.84%) of the screened individuals was included in non-CRVO group. In **PART II**, of 45 patients in CRVO group, 5 (11.11%) patients were excluded for follow-up duration of <1 year. Finally, 40 patients (88.89%) were included in the final comparative analysis, with 15 in non-HROD group and 25 in HROD group.

A total of 45 eyes (24 right eyes vs 21 left eyes) from 45 patients (21 males vs 24 females) were enrolled in the CRVO group and 63 eyes from another 63 patients (33 males vs 30 females) in the non-CRVO group, without statistical significance in male percentage (46.67% vs 52.38%, *p* = 0.558), age distribution (64.16 ± 12.05 vs 60.03 ± 14.00, *p* = 0.113), primary glaucoma (11.11% vs 4.76%, *p* = 0.385), and diopters before intraocular surgery (−0.33 ± 1.26 vs −1.13 ± 1.82, *p* = 0.289), but with a significant statistical difference in HROD (51.16% vs 26.98%, *p* = 0.010).

In the comparison of metabolic disorder(s), there were significant differences in hypertension percentages (57.78% vs 79.37%, *p =* 0.015), maximum SBP (150.66 ± 21.56 vs 163.07 ± 26.00, *p* = 0.013), but no significant differences in diabetes percentages (*p =* 0.113), diabetes duration (*p* = 0.525), hypertension grade (*p* = 0.301), hypertension duration (*p* = 0.446), maximum diastolic blood pressure (*p* = 0.110), and pulse pressure difference (*p* = 0.052) and dyslipidemia percentages (*p* = 0.971).

**FIGURE 4:**
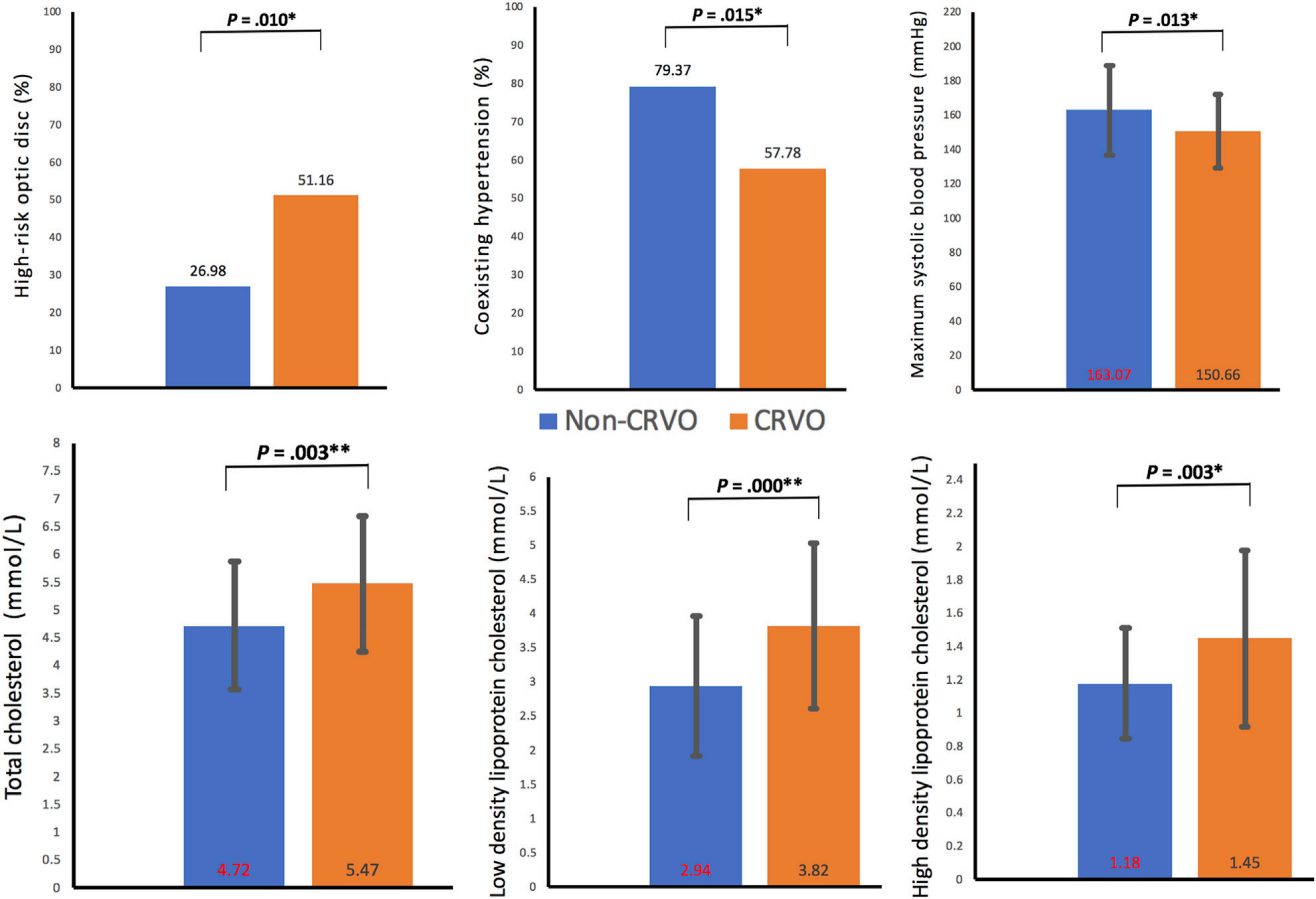
Clinical characteristics with statistical differences between the two groups. CRVO, central retinal vein occlusion. *p < 0.05; **p < 0.01.

In the comparison of blood tests, there were significant statistical differences between the mean values of TC (5.47 ± 1.21 vs 4.72 ± 1.15, *p* = 0.003), LDL.C (3.82 ± 1.21 vs 2.94 ± 1.02, *p* = 0.000), and HDL-C (1.45 ± 0.53 vs 1.18 ± 0.33, *p* = 0.003). The comparison of other blood variables showed no statistical difference, including hemoglobin A1c, triglyceride, and eGFR (*p =* 0.744, 0.261, and 0.730, respectively). The demographic, ocular, and systemic characteristics of the recruited patients with metabolic disorder(s) were summarized in Table 1 and depicted in Figure 4.

**TABLE 1:** Demographic, ocular and systemic characteristics of all recruited patients with metabolic disorder(s).

Baseline characteristics	All N = 108	Grouping		*p*-Value
		Non-CRVO (N = 63)	CRVO (N = 45)	
Demographic and ocular features				
Male	54 (50.00)	33 (52.38)	21 (46.67)	0.558
Age, y	61.75 ± 13.32	60.03 ± 14.00	64.16 ± 12.05	0.113
Right eye ^&&^	-	-	24 (53.33)	1.000
HROD	39 (36.11)	17 (26.98)	22 (51.16)	0.010*
Coexisting primary glaucoma	8 (7.41)	3 (4.76)	5 (11.11)	0.385
Diopters before intraocular surgery, D	−1.13 ± 1.82	−1.13 ± 1.82	−0.33 ± 1.26	0.289
Metabolic disorders				
Coexisting diabetes	73 (67.60)	47 (74.60)	26 (57.78)	0.113
Coexisting dyslipidemia	77 (71.30)	45 (71.43)	32 (71.11)	0.971
Coexisting diabetes and dyslipidemia	42 (38.89)	29 (46.03)	13 (28.89)	0.072*
Diabetes duration, y	6.77 ± 7.19	7.15 ± 6.88	6.23 ± 7.67	0.525
Coexisting hypertension	76 (70.37)	50 (79.37)	26 (57.78)	0.015*
Hypertension grade, median (IQR) &	1 (1, 2)	2 (1, 3)	1 (1, 2)	0.301
Hypertension duration, y, median (IQR) &	2 (1, 10)	2 (1, 10)	2 (0, 7.75)	0.446
Maximum systolic blood pressure, mmHg	157.56 ± 24.80	163.07 ± 26.00	150.66 ± 21.56	0.013*
Maximum diastolic blood pressure, mmHg	89.43 ± 14.20	91.50 ± 14.03	86.89 ± 14.15	0.110
Pulse pressure difference, mmHg	67.93 ± 20.72	71.59 ± 21.00	63.43 ± 19.68	0.052
Blood tests				
Hemoglobin A1c, %	6.77 ± 1.10	6.79 ± 1.04	6.69 ± 1.34	0.744
TC, mmol/L	5.02 ± 1.22	4.72 ± 1.15	5.47 ± 1.21	0.003*
Triglyceride, mmol/L	1.71 ± 1.02	1.81 ± 1.05	1.57 ± 0.97	0.261
LDL.C, mmol/L	3.29 ± 1.17	2.94 ± 1.02	3.82 ± 1.21	0.000**
HDL.C, mmol/L	1.29 ± 0.44	1.18 ± 0.33	1.45 ± 0.53	0.003*
eGFR, mL/min/1.73m^2^	87.20 ± 21.67	86.74 ± 22.52	88.81 ± 18.89	0.730

CRVO = central retinal vein occlusion; eGFR = estimated glomerular filtration rate; HbA1c = glycated hemoglobin A1c; HDL.C = high density lipoprotein cholesterol; HROD = high risk optic disc; IQR = Interquartile range; IOL = intraocular lens; LDL.C = low density lipoprotein cholesterol; TC = total cholesterol; No hypertension = hypertension grade 0. Primary glaucoma = primary open-angle glaucoma or primary angle-closure glaucoma. * = *p* < 0.05; ** = *p* < 0.01.

Data were presented as mean ± standard deviation, median (IQR) or no. (%).

& Mann-Whitney U test was used to compare the statistical differences between two groups; && McNemar test.

*p* < 0.05 was considered to be statistically significant.

### Spearman rank-based correlation and pearson’s partial correlation for CRVO

In Spearman rank-based correlation analysis between all baseline features and CRVO, only HROD, maximum SBP, TC, LDL.C and HDL.C showed significant correlations with correlation coefficients of 0.257 (*p* = 0.007), −0.231 (*p* = 0.021), 0.291 (*p* = 0.004), 0.358 (*p* = 0.000) and 0.312 (*p* = 0.002). Pearson's partial correlation analysis showed that only LDL.C (*adjusted p* = 0.012) was significantly associated with CRVO. Detailed correlation analyses for CRVO were summarized in Table 2.

**TABLE 2:** Correlation analysis for CRVO.

Ocular and systemic features (N = 108)		Spearman rank-based correlation for CRVO		
		Coefficient	P *	Adjusted P **
HROD (n, %)	39 (36.11)	0.257	**0.007**	**0.067**
Coexisting diabetes and dyslipidemia (n, %)	42 (38.89)	−0.239	0.019	0.469
Coexisting hypertension (n, %)	76 (70.37)	−0.139	0.168	0.412
Maximum systolic blood pressure, mmHg	157.56 ± 24.80	−0.231	**0.021**	0.335
Pulse pressure difference, mmHg	67.93 ± 20.72	−0.191	0.059	0.444
TC, mmol/L	5.02 ± 1.22	0.291	**0.004**	0.474
LDL.C, mmol/L	3.29 ± 1.17	0.358	**0.000**	**0.012**
HDL.C, mmol/L	1.29 ± 0.44	0.312	**0.002**	0.574

CRVO = central vein occlusion; HDL.C = high-density lipoprotein cholesterol; HROD = high risk optic disc; LDL.C = low-density lipoprotein cholesterol; SD = standard deviation; TC = total cholesterol.

* Spearman rank-based correlation analysis; ** Pearson’s partial correlation analysis. Significant *p* values (*p* < 0.05) are in bold font.

### The univariable and multivariable logistic regression analysis for CRVO

In univariable regression analysis for CRVO vs non-CRVO group, HROD (OR = 2.964, 95% CI 1.316–6.676, *p =* 0.009) and LDL.C (OR = 2.033, 95% CI 1.349–3.066, *p* = 0.001) were significantly associated with CRVO. And then, in further multivariate regression analysis, HROD (OR = 5.036, 95% CI 1.847–13.729, *p* = 0.002) and LDL.C (OR = 2.311, 95% CI 1.458–3.663, *p* = 0.000) were both independent risk factor. Univariate and multivariate logistic regression analyses for CRVO were summarized in Table 3.

**TABLE 3:** The univariable and multivariable logistic regression analysis for CRVO.

Risk factors	Univariable logistic regression			Multivariable logistic regression		
	OR	95% CI	*p*-Value	OR	95% CI	*p*-Value
HROD						
CRVO vs non-CRVO	2.964	1.316–6.676	0.009*	5.036	1.847–13.729	0.002*
LDL.C, per mmol/L increase						
CRVO vs non-CRVO	2.033	1.349–3.066	0.001*	2.311	1.458–3.663	0.000**

CI, confidence interval; CRVO, central retinal vein occlusion; HROD, high risk optic disc; LDL.C, low-density lipoprotein cholesterol; OR, odds ratio. * = P < 0.05; ** = P < 0.001

P < 0.05 was considered to be statistically significant.

### Comparison of demographic, treatment and prognosis between CRVO patients with and without HROD

Among 40 CRVO eyes followed up for ≥1 year, the independent-sample *t*-test showed no significant difference in comparison of male percentage (46.67% vs 40.00%, *p* = 0.918), age distribution (62.27 ± 15.61 vs 61.88 ± 11.74, *p* = 0.996), and right laterality percentage (66.67% vs 44.00%, *p* = 0.372) between the two groups of CRVO eyes (non-HROD group, N = 15, and HROD group, N = 25, respectively), as well as in comparison with CWTBRAs, hard exudation around macular, ischemic CRVO (*p =* 0.938, 0.988, and 0.735, respectively). In addition, detailed follow-up information, including treatment and prognosis of the affected eyes, showed no statistical differences (all *p* > 0.05). All follow-up parameters included follow-up duration, interval from CRVO onset to first IVT, IVTs of anti-vascular endothelial growth factor medicine, IVTs of dexamethasone implant, total number of IVTs, retinal photocoagulation treatment, IOL implantation, OHT event within follow-up duration, BCVA at first visit, 1-month BCVA after first IVT, lowest central retinal thickness within follow-up duration, BCVA at the final visit, low vision, blindness, and NVG. All parameters, calculation results, and *p* values used for comparison between the two groups of eyes were shown in Table 4.

**TABLE 4:** Comparison of demographic, follow-up information and prognosis between CRVO eyes with and without HROD.

Variables	All (N = 40)	Grouping		*p*-Value
		Non-HROD (N = 15)	HROD (N = 25)	
Baseline demographic and laterality				
Male	17 (42.50)	7 (46.67)	10 (40.00)	0.918
Age, y	62.03 ± 13.13	62.27 ± 15.61	61.88 ± 11.74	0.996
Right eye	20 (50.00)	10 (66.67)	11 (44.00)	0.372
Characteristics of CFP at last visit				
CWTBRAs	12 (30.00)	5 (33.33)	7 (28.00)	0.938
Hard exudation around macular	3 (7.50)	1 (6.67)	2 (8.00)	0.988
Ischemic CRVO	13 (32.50)	6 (40.00)	7 (28.00)	0.735
Follow-up information of affected eyes				
Follow-up duration, m	16.47 ± 15.79	16.08 ± 10.91	16.68 ± 18.32	0.995
Interval from onset to first IVT, m	4.63 ± 14.21	1.38 ± 1.30	6.70 ± 17.99	0.550
Number of anti-VEGF medicine IVTs	3.89 ± 3.62	4.23 ± 3.94	3.71 ± 3.51	0.919
Number of DEX implant IVTs	0.84 ± 1.54	0.77 ± 1.64	0.88 ± 1.51	0.980
Total number of IVTs	4.73 ± 4.03	5.00 ± 4.08	4.58 ± 4.09	0.957
Retinal photocoagulation treatment	9 (22.50)	5 (33.33)	4 (16.00)	0.704
IOL implantation	14 (35.00)	7 (46.67)	7 (28.00)	0.488
OHT event within follow-up duration	4 (10.00)	1 (6.67)	3 (12.00)	0.862
BCVA at first visit, log MAR	0.87 ± 0.55	0.66 ± 0.48	0.99 ± 0.56	0.211
1-month BCVA after first IVT, log MAR	0.74 ± 0.61	0.67 ± 0.59	0.78 ± 0.63	0.891
LCRT within follow-up duration, μm	194.21 ± 39.83	198.64 ± 36.84	190.95 ± 42.58	0.862
Prognosis				
BCVA at final visit, log MAR	1.03 ± 0.93	0.85 ± 0.94	1.13 ± 0.92	0.657
Low vision	21 (52.50)	7 (46.67)	14 (56.00)	0.878
Blindness	14 (35.00)	5 (33.33)	9 (36.00)	0.985
NVG	8 (20.00)	3 (20.00)	5 (20.00)	1.000

BCVA = the best corrected visual acuity; CFP = color fundus photography; CRVO = central retinal vein occlusion; CWTBRAs: complete whiteness of 1 or 2 temporal branch retinal arteries; DEX = dexamethasone; HROD = high-risk optic disc; IOL = intraocular lens; IVT = intravitreal treatment; LCRT = lowest central retinal thickness; log MAR = logarithm of the minimum angle of resolution; NVG = neovascular glaucoma; OHT = ocular hypertension

Data were presented as mean ± standard deviation or no. (%)

*p* < 0.05 was considered to be statistically significant

## Discussion

In our study, a statistically significant difference in the HROD percentage (51.16% vs 26.98%, *p* < 0.05) was found between the CRVO and non-CRVO groups. In the univariable logistic regression analysis, both HROD (OR = 2.964, 95% CI 1.316–6.676, *p* = 0.009) and LDL.C (OR = 2.033, 95% CI 1.349–3.066, *p* = 0.001) were shown to be risk factors for CRVO, with the non-CRVO group as a reference. After excluding the influence of other potential risk factors (including hypertension, diabetes, TC, and coexisting primary glaucoma, etc.), in further multivariate logistic regression analysis, both HROD (OR = 5.036, 95% CI 1.847–13.729, *p* = 0.002) and LDL.C (OR = 2.311, 95% CI 1.458–3.663, *p* = 0.000) became the independent risk factors for CRVO. Therefore, our research revealed that both HROD and LDL.C were independent risk factors for CRVO occurrence in individuals with metabolic disorder(s). In the individuals with metabolic disorder(s), compared with the eyes without HROD, the risk of developing CRVO in the eyes with HROD was 4.036 times higher; And for every 1 mmol/L increase in LDL.C, the risk of developing CRVO would increase by 131.1%.

In previous studies (Mansour et al., 1990; Xu et al., 2012), there has been controversy over the correlation between HROD and CRVO, indicating that HROD may not be an independent risk factor in the overall individuals. However, in specific individuals with metabolic disorder(s), both HROD and LDL.C were independent risk factors for CRVO occurrence. We speculated that patients with metabolic disorder(s) were already in a hypercoagulable state, and the crowded optic disc further exacerbated venous hypostasis in central retinal vein, ultimately leading to CRVO occurrence. Similarly, Lei et al. (2021) also found that HROD was more common among female children with CRVO. This research also indicated that HROD increased the risk of CRVO occurrence in specific individuals of female children. Therefore, it is of great significance to explore the specific risk of HROD on CRVO occurrence in different individuals.

As reported in the literature (Elman et al., 1990; Alduaij et al., 2018; Wang et al., 2020; Simmons et al., 2022), diabetes was also a risk factor for CRVO with an OR value of 1.98 (OR = 1.98, 95% CI:1.29–3.03, I^2^ = 67.9%) (Wang et al., 2020) and coexisting diabetes accounted for 13%–34% of patients with CRVO (Alduaij et al., 2018). Among our subjects with CRVO, the proportion of coexisting diabetes was 57.78%, which was higher than the above literature, and was also higher than 24% by Demir S et al. (2015) and 16.7% by Christodoulou et al. (2019) However, Pinna et al. believed that diabetes might be a protective factor for branch retinal vein occlusion (Pinna et al., 2007). Therefore, diabetes had a potential risk for CRVO occurrence. In order to eliminate this potential risk factor, we selected the non-CRVO control group matched by blood glucose levels. In this study, we focused on the influencing factors for CRVO in patients with metabolic disorder(s), and excluded a large number of patients without metabolic disorder(s) when entering the CRVO group. As a result, the proportion of diabetes patients in CRVO group of this study was relatively high, but there was no statistical difference between the CRVO and non-CRVO groups. Not only that, we also matched gender and age to eliminate the potential impact of demographic factors on RVO. It had been reported that RVO was more common in individuals over 65 years of age (Hayreh et al., 2001; Hayreh et al., 2002; Kolar, 2014), with the mean age (in 2001) of (56.9 ± 10.2) years (Kolar, 2014), which was lower than our reported average age of (64.16 ± 12.05) years in the CRVO group.

As mentioned above, in patients with metabolic abnormalities, with the non-CRVO group as a reference, HROD was an independent risk factor for the CRVO occurrence with an OR value of 5.036. In our study, the specific risk did not change with the coexisting primary glaucoma, and coexisting hypertension and dyslipidemia. At baseline comparison, the proportion of glaucoma in both groups was relatively small, and there was no statistically significant difference between the two groups, which basically ruled out the impact of coexisting primary glaucoma on CRVO occurrence. In terms of coexisting hypertension analysis, although there were statistical differences in the proportion of coexisting hypertension proportion and maximum systolic blood pressure values between the two groups at baseline (*p* = 0.015, 0.013, respectively). Further Pearson’s partial correlation analysis showed that they were not independent factors related to CRVO occurrence (adjusted *p* = 0.412, 0.226, respectively). Therefore, the baseline characteristics of hypertension did not affect the accuracy of the study results. In addition, our study also investigated the impact of baseline blood lipid levels on CRVO occurrence. Although the CRVO group had higher TC, LDL.C, and high-density lipoprotein cholesterol, compared to the non-CRVO control group at baseline (*p* = 0.003, 0.000, and 0.003, respectively). LDL.C was usually highly correlated with TC and HDL.C, which was also reflected in our study. However, further Pearson’s partial correlation analysis showed that apart from LDL.C (adjusted *p* = 0.013), neither TC nor high-density lipoprotein cholesterol (adjusted *p* = 0.396, 0.511, respectively) were independent correlated factors for CRVO occurrence. On the contrary, the correlation between them and CRVO was affected by LDL.C, so they were not included in the multivariable logistic regression analysis.

Also, we explored the impact of laterality on CRVO. In fact, research on the laterality preference of CRVO is very rare, and only a recent article mentioned that CRVO occurred slightly more frequently in the left eye (Li et al., 2022). However, in our eye laterality preference study, and we did not find the laterality preference (53.33% vs 46.67%, *p* = 1.000, McNemar test). A possible explanation is that the anatomical characteristics of the aortic arch resulted in higher perfusion pressure (PP) of the right internal carotid artery and a lower PP of the left internal carotid artery (Malone et al., 2019); Subsequently, the PP of the left ocular artery was relatively low. The relatively low PP coupled with increased venous reflux resistance caused by HROD (Beaumont and Kang, 2002), ultimately increased the risk of CRVO in the left eye.

Finally, we discussed the potential protective factors of CRVO. Numerous studies have observed protective factors of RVO, including blood high-density lipoprotein cholesterol by Pan et al. (2022), coexisting glucose-6-phosphate dehydrogenase deficiency by Pinna et al. (2007), adrenomedullin-Receptor Activity-Modifying Protein 2 System by Hirabayashi et al. (2019), retinochoroidal collateral veins by Fuller et al. (2003), activated protein C by Du et al. (2011) and oral pentoxifylline by Park et al., 2007. However, none of the potential risk factors we screened were protective (including blood high-density lipoprotein cholesterol, adjusted *p* = 0.511, Pearson’s partial correlation analysis).

In summary, HROD is likely to be an independent risk factor for CRVO occurrence in individuals with metabolic disorder(s). There was no statistical difference in the treatment and prognosis of CRVO between the eyes with and without HROD. However, this study had some limitations. This was a retrospective, non-randomized case-control clinical study, and the results might have been affected by selective bias, especially in the selection of the non-CRVO group. In addition, we did not conduct a further quantitative analysis of the parameters of HROD; therefore, we could not confirm the minimum volume limit of the optic cup that would increase the risk of CRVO. Third, all enrolled patients were Chinese Han people, so it was not clear whether our conclusions apply to other races or nationalities. We will continue to address these limitations in the future.

## Data Availability

The raw data supporting the conclusions of this article will be made available by the authors, without undue reservation.

## Abbreviations

BCVA, Best-corrected visual acuity; CFP, color fundus photography; CRVO, central retinal vein occlusion; CWTBRAs, complete whiteness of one or two temporal branch retinal arteries; eGFR, estimated glomerular filtration rate; HbA1c, glycated hemoglobin A1c; HROD, high-risk optic disc; IQR, interquartile range; IVT, intravitreal treatment; log MAR, logarithm of the minimum angle of resolution; NVG, neovascular glaucoma; OHT, ocular hypertension; OCT, optical coherence tomography; ORs, odds ratios; PP, perfusion pressure; RVO, retinal vein occlusion; CRVO-ME, central retinal vein occlusion related macular edema; VCD, Rvertical cup-to-disc ratio.
